# FEASIBILITY OF A COMMUNITY-LED VIRTUAL SELF-CARE PROGRAM FOR AFRICAN AMERICAN FAMILY CAREGIVERS

**DOI:** 10.1093/geroni/igac059.2547

**Published:** 2022-12-20

**Authors:** Abiola Keller, Nia Norris, Bashir Easter, Andrea Garr, Gail Morgan, Ramona Dicks-Williams, Colleen Galambos

**Affiliations:** Marquette University, Milwaukee, Wisconsin, United States; UW-Madison Wisconsin Alzheimer's Institute Regional Milwaukee Office, Milwaukee, Wisconsin, United States; University of Wisconsin Madison, Madison, Wisconsin, United States; Wisconsin Department of Health Services, Milwaukee, Wisconsin, United States; UW Madison School of Medicine and Public Health, Milwaukee, Wisconsin, United States; Community Member, Milwaukee, Wisconsin, United States; University of Wisconsin-Milwaukee, Milwaukee, Wisconsin, United States

## Abstract

As the population of older adults in the U.S. increases, the need for family caregivers will mirror this growth. Despite a greater intensity of caregiving, African American caregivers are less likely to access formal support services and engage in self-care practices. To address the self-care needs of African American family caregivers, a community-engaged approach was used to develop and implement a half-day virtual self-care program. The development of the program was guided by the Individual and Family Self-Management Theory. This study evaluates experiences of program attendees. The specific aims are to examine the acceptability and practicality of the virtual self-care program. Women who self-identified as African American and a caregiver (i.e., taking care of another adult) were personally invited to attend. All attendees were emailed an electronic survey containing Likert-type and open-ended questions. Responses were examined for patterns and key content-related categories using inductive content analysis. Eleven of the sixteen attendees responded to the survey. All eleven strongly agreed (64%) or agreed (36%) that the event met their expectations and/or needs. In addition to providing opportunity to take time to engage in self-care, the event created a virtual space for women to focus on themselves. Women spoke about three distinct ways the event met their needs: 1) learning and trying new things, 2) access to resources, and 3) having a shared experience. These findings suggest that virtual programs may be used as an additional resource to support the health of African American women who care for older adults.

